# Healthcare Interventions to Support Informal Caregivers of People With Severe Mental Illnesses: A Scoping Review

**DOI:** 10.1111/inm.70236

**Published:** 2026-02-11

**Authors:** Marjolijn Heslinga, Marie Louise Luttik, Bouwina Esther Sportel, Evelyn Finnema, Nynke Boonstra

**Affiliations:** ^1^ Health Science‐Nursing Science and Education University Medical Center Groningen Groningen the Netherlands; ^2^ Research Group Nursing Diagnostics and Family Care Hanze University of Applied Sciences Groningen the Netherlands; ^3^ Department of Psychotic Disorders GGZ Drenthe Mental Health Institute Assen the Netherlands; ^4^ NHL Stenden University of Applied Sciences Leeuwarden the Netherlands; ^5^ Department of Psychiatry University Medical Center Utrecht, University of Utrecht Utrecht the Netherlands; ^6^ KieN VIP Mental Health Care Services Leeuwarden the Netherlands

**Keywords:** family support, informal caregiver, mental health, nursing, psychosocial interventions

## Abstract

In recent years, care for people with severe mental illnesses (SMI) has shifted from institutional to recovery‐based treatment in which informal caregivers often have an important role. However, their increased involvement has elevated issues such as role strain or burden of care, which indicates that they need greater support. Adding to existing reviews, this study is intended to provide an overview of interventions that support informal caregivers who are tending to people with severe depression, anxiety or personality disorders other than borderline personality disorder (BPD). A scoping review, following the JBI guidelines, was performed. Pubmed, PsycInfo, CINAHL, Embase and Cochrane Library were searched using the keywords ‘caregiver’, ‘SMI’, ‘support’, ‘interventions’ and related terms, which led to 21 720 references. After removing duplicates (*n* = 9043) and screening titles, abstracts (*n* = 12 677) as well as full‐text (*n* = 108) for inappropriate results, 13 articles met the inclusion criteria and are included in this review. The included articles describe 11 different interventions. Interventions are built on diverse concepts or mechanisms, clustered in: ‘changing thoughts and behaviour’, ‘understanding the illness’ and ‘feeling supported’. Interventions described in this review are similar to those outlined in previous reviews that focus on, for example, psychotic disorders, and could decrease informal caregiver's burden and strain. Considering that only 13 relevant articles were identified from our search, we emphasise the need for further scientific attention to explore interventions that support the informal caregivers of people with depression, anxiety or personality disorders. Future researchers should explore how various intervention components and contextual factors affect outcomes, meet caregiver's diverse needs, and support practical implementation.

## 1 Introduction

Approximately one in eight people globally live with a mental disorder (WHO 2022). This comprises approximately 970 million people, of whom one quarter are dealing with a severe mental illness (SMI). SMI, also described as severe mental disorder (SMD), is defined as a psychiatric disorder that substantially limits social functioning and requires long‐term coordinated treatment (Delespaul 2013). According to the *Diagnostic and Statistical Manual of Mental Disorders, Fifth Edition* (DSM‐5), diagnostic spectra that meet the definition of SMI include, for example, psychotic, bipolar, depressive, personality and anxiety disorders (Delespaul 2013).

Over the past few decades, the long‐term care of people with SMIs has shifted from institutional care to community mental healthcare (EUROSTAT 2020) for several reasons. First, a recovery concept was introduced that focuses on the wants and needs of people with SMI and seeks to organise services for these people (Anthony 1993). This led to, among other things, increased attention on patient wishes and social participation (Vanner and Keet 2019). De‐institutionalising long‐term psychiatric care increased the number of patients with SMI who receive community healthcare while also encouraging informal caregivers' involvement in the care of psychiatric patients.

The second reason for the growing participation of informal caregivers is the rising health‐care costs for chronic diseases. European health care policy is focused on community‐based care, which with the collaboration of informal caregivers, such as family members, neighbours or significant others, may not only improve the quality of care for people with mental disorders but also decrease governmental health‐care costs (Busse et al. 2010; Spasova et al. 2018; WHO 2008). The third reason is the evidence of informal caregiver's positive influence on recovery outcomes and the self‐management strategies of people with SMI (Lean et al. 2019; Pharoah et al. 2010; Reinares et al. 2016).

As informal caregiver's role increases in caring for patients with SMI, potential problems with family burdens grow (Adelman et al. 2014). Researchers report a significant level of family burden and conflict among informal caregivers for people with, for example, bipolar disorder (Reinares et al. 2016) and first episode psychosis (Dillinger and Kersun 2020). To prevent or ease the burden of care on families, nurses and other professionals need to support informal caregivers, facilitate collaboration (Adelman et al. 2014), use supportive interventions (Hansen et al. 2022) and invite informal caregivers to partner with the treatment team (Adelman et al. 2014; Anker‐Hansen et al. 2018).

Several reviews regarding the support for informal caregivers for people with SMI have already been performed. A systematic review of informal caregiver‐focussed interventions indicates that education and support may improve the caregiving experience and quality of life while decreasing the burden and psychological distress of people who are caring for patients with SMI (Yesufu‐Udechuku et al. 2015). Another review on the effect of mental health interventions for informal caregivers for people with mental illness reveals a positive effect of decreasing informal caregivers' psychological distress through interventions such as psycho‐education, psychosocial, multicomponent or cognitive behavioural therapy (CBT) and mindfulness‐based as well as support group interventions (Hansen et al. 2022). Finally, two reviews that have focussed on supportive interventions for caregivers for people with borderline personality disorder (BPD) have demonstrated positive effects on carer's burden (Guillén et al. 2020; Sutherland et al. 2019).

Although these reviews provide important information about supportive interventions for informal caregivers, they include only randomised controlled trials (RCTs) or quasi‐experimental designs and describe either a specific population, such as those with psychotic and bipolar disorders (Yesufu‐Udechuku et al. 2015), BPD (Guillén et al. 2020; Sutherland et al. 2019) or a broad population, including children and those with dementia (Hansen et al. 2022). None of the existing reviews to date examine the described supportive interventions in studies that are broader than RCTs or address SMIs other than psychotic disorder, bipolar disorder and BPD. Community‐based healthcare is used with an extensive patient population in which DSM‐5 diagnostics gradually change or may be comorbid (Delespaul 2013); hence, it is important to ensure that supportive interventions are usable and integrated within the full scope of SMI.

## 2 Aims

This study initially aimed to provide an overview of supportive interventions within the full scope of SMI. However, during the research process we found several recent reviews about psychotic and bipolar disorders and BPD, leading to a narrowed focus to SMI diagnoses receiving less attention in literature reviews. Therefore, this study provides an overview of interventions described in the scientific literature that support informal caregivers for people who have SMI and have been diagnosed with a depressive or anxiety disorder or a personality disorder other than BPD. Since various professionals are involved in community‐based treatment (e.g., nurses, psychiatrists, psychologists, social workers), we include supportive interventions applied by all kinds of professionals.

## 3 Method

### 3.1 Design, Protocol and Registration

A scoping review of literature was conducted, as the aim of this study is to map the interventions that are described in various types of scientific research (Arksey and Malley 2005; Peters et al. 2015) and are employed by a heterogeneous group of healthcare professionals (Peters et al. 2015).

The review was guided by the Joanna Briggs Institute (JBI) Manual for Evidence Synthesis (Peters et al. 2015). To facilitate complete, transparent reporting and to improve the research quality, the PRISMA‐ScR checklist (Tricco et al. 2018) was used. The study protocol was registered in the Open Science Framework (https://doi.org/10.17605/OSF.IO/KDU5N).

### 3.2 Eligibility Criteria

A broad literature search following the initial study protocol was performed in February 2024, with an update in June 2025. Articles focusing on adult patients with SMI and the support of their families or informal caregivers in both inpatient and outpatient contexts were initially included. We initially accepted articles that were published since 1993 in English in any type of research publication (quantitative, qualitative and review publications).

Articles were excluded if they focussed on informal caregivers for patients with congenital brain injury, dementia, mental problems induced by pregnancy or during postpartum period, or primary physical problems; we also excluded articles focussing on informal caregivers for children (< 18 years). Based on our study protocol, this search led to 422 included articles. Therefore, we added for both practical and substantive reasons, exclusion criteria to our search: studies that focus on psychotic or bipolar disorders and BPD were excluded because of the multiple reviews that have addressed these specific disorders (Chen et al. 2016; Dillinger and Kersun 2020; Guillén et al. 2020; Lobban et al. 2013; Sutherland et al. 2019; Yesufu‐Udechuku et al. 2015). Articles regarding autism spectrum disorders (ASD) were excluded since ASD is a developmental disability or spectrum disorder that has a wide variation in type or severity (National Institute of Mental Health 2024), and articles about substance use disorders were excluded, as these disorders are often comorbid with other conditions. Finally, studies published before 2014 were excluded. No articles were excluded based on design or quality assessment.

### 3.3 Information Sources, Search Strategy and Screening Process

Pubmed, PsycInfo, Cumulative Index of Nursing and Allied Health (CINAHL), Embase, and Cochrane Library were searched by using keywords that were determined after consultation with a librarian (see Table 1). For the complete search strategy, refer to Appendix S1.

**TABLE 1:** Search terms.

Content area~	Subject heading~	Search terms~			
Caregiver	Family Caregiver Relative	Family^a^ Caregiver^a^ Spouse^a^	Relative^a^ Dyad^a^ Husband^a^	Wife^a^ Sibling^a^ Brother^a^	Sister^a^
SMI	Mental Illness Bipolar disorder Psychotic disorder Depressive disorder Anxiety disorder Substance‐related disorder Personality disorder Autism spectrum disorder	Mental illness^a^ Mental disorder^a^ Bipolar disorder^a^ Schizophren^a^ Psychot^a^ Psychotic disorder^a^	Depres^a^ Depressive disorder^a^ Anxiet^a^ Anxiety disorder^a^	Substance‐related disorder^a^ Addict^a^ Personality disorder^a^ Forensic^a^	Autistic Autism^a^ Autism spectrum disorder
Support	Support	Support^a^			
Interventions	Intervention	Psychosocial intervention^a^	Intervention^a^	Train^a^ ^y^	

*Note:* ~ Boolean methods AND and OR were used.

^a^ Truncation was employed to retrieve relevant records by capturing word variants with different endings.

The initial search yielded 18 747 references, and a search update in June 2025 yielded another 2973 references. Thus, 21 720 references were imported into Rayyan (Ouzzani et al. 2016) and 9043 duplicate articles were removed. To ensure reliable data collection, one‐third of 12 677 articles were screened by minimally two reviewers who screened articles by title and abstract. Screening was based on inclusion and exclusion criteria, as well as on indicators of SMI (limitations in social functioning or description of long term treatment) within the described samples. During the whole screening process, the research team discussed all conflicts or doubts about inclusion and exclusion, and regarding the definition of SMI. This discussion was followed by the decision for in or exclusion. The remaining two‐thirds of the search were screened by one reviewer (MH or BES). Potentially relevant articles were included or excluded through consensus; discrepancies were resolved by a third reviewer (MLL). A total of 12 145 were excluded and 108 articles were selected for full‐text screening.

**FIGURE 1:**
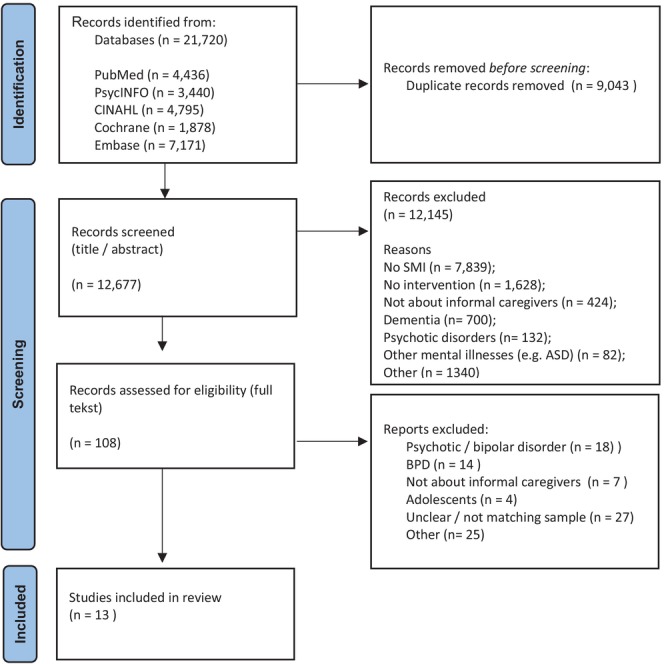
Prisma flowchart based on search.

The full‐text articles were assessed for eligibility by one reviewer (MH) (see Figure 1) and potential relevant articles were discussed with the other two reviewers (MLL, BES). Articles (*n* = 95) were excluded for the following reasons: focus on psychotic or bipolar disorders (*n* = 18) or BPD (*n* = 14), focus on the patient rather than on the informal caregiver (*n* = 7), or focus on people under 18 years of age (*n* = 4). Seven articles provided broad or unclear information about patient diagnostics; further enquiry led to three responses, which were then excluded based on population. The other four enquiries received no response, which also led to exclusion. Twenty articles with a mixed sample in patient diagnostics were excluded since less than 50% of the patients in the described sample met the inclusion criteria. Finally, 25 articles were excluded for reasons such as being a protocol paper or being written in languages other than English.

Both the initial search and its subsequent update resulted in the identification of 13 relevant articles for this review.

The PRISMA flow diagram (Figure 1) illustrates the selection process (Peters et al. 2015).

### 3.4 Data Charting Process and Data Items

A data extraction tool was generated to chart, analyse and present the data. The data charting process was performed by the first author (MH), and each step was discussed with the research team. Data was extracted on the following predefined characteristics: intervention name, author and year of publication, country in which the research was performed, design (including level of evidence), target population and type of caregiver, context/setting, and professional involved in the intervention (see Table 2).

**TABLE 2:** Included articles (*n* = 13) and described interventions (*n* = 11).

Intervention name	Author and year of publication	Country	Research design and level of evidence^a^	Intervention target population and type of caregiver	Context or setting	Professional involved in the intervention	Measurements
Compassion cultivation training programme (CCT)	Holland Hansen et al. (2021)	Denmark	RCT (2)	Caregivers of people with mental illnesses (mean age 53 years)	Community setting	CCT trainer	*Quantitative* Psychological distress: Depression Anxiety Stress Scale (DASS) Stress: Perceived Stress Scale (PSS) Resilience: Brief Resilience Scale Well‐being: World Health Organization‐ Five Well‐Being Index Emotion regulation: Emotion Regulation Questionnaire Self‐compassion: The 12‐item Self‐Compassion Scale Short Form (SCS‐SF) Dimensions of compassion: Multidimensional Compassion Scale Dimensions of mindfulness: Five Facet Mindfulness Scale
Dialectic behavioural treatment (DBT) skill group	Wilks et al. (2017)	United States	Quasi‐experimental design (3)	Family members of individuals with behavioural disorders (mean age 55 years)	Community setting	Graduate students and postdoctoral therapists	*Quantitative* Interpersonal problems: Inventory of Interpersonal Problems‐Personality Disorders 25 (IPP‐PD‐25) Attitudes towards family relationship: Family Attitude Scale (FAS) Caregiver strain: Caregiver Strain Questionnaire‐Short Form 7 (CGSQ‐SF7) Emotion dysregulation: Difficulties in Emotion Regulation Scale (DERS) Depression: Patient Health Questionnaire‐9 (PHQ‐9) Anxiety: State Trait Anxiety Inventory (STAI) Stress: Perceived Stress Reactivity Scale (PSRS)
E‐care for caregivers	Bijker et al. (2017)	The Netherlands	Mixed‐method design comprising a pilot RCT and qualitative interview (3)	Nonprofessional adult caregivers (partners, parents, children, siblings, family or friends of depressed patients) (aged 21–85 years)	Variety (no treatment, primary, secondary care or during admission)	Cognitive behavioural therapist and caregiver peers	*Quantitative* Usability: System Usability Scale (SUS) Psychological distress: Kessler‐10 Anxiety symptoms: Generalised Anxiety Disorder Scale (GAD‐7) Subjective burden: Zarit Burden Interview (ZBI) Quality of life: EuroQol Five Dimensions Health Questionnaire (EQ5D) Mastery: Pearlin Mastery (PM) Scale *Qualitative* User‐friendliness (in addition to SUS): Semi‐structured telephone interviews
Family‐centred support conversations (FCSC)	Aass et al. (2020)	Norway	Qualitative phenomenological design (6)	Family (self‐identified as family) of young adults with mental illness (aged 20–55 years)	Community setting	Mental health care professionals (nurse, social worker and social educator)	*Qualitative* FCSC experience: interview
Family recovery peer support (FRPS)	Reynolds et al. (2022)	Ireland	Qualitative phenomenological design (6)	Family members of people with SMI (aged 23–69 years)	Unknown	Peers	*Qualitative*: Experience with a family member who suffers from mental illness and experience of receiving FRPS: interview
Guided self‐help (GSH)	McCann et al. (2015a, 2015b)	Thailand	RCT (2) RCT (2)	Family members of people with depression (mean age 41 years)	Outpatient treatment	Self‐help with support researcher	*Quantitative* McCann et al. (2015a) Resilience: Resilience Scale (RS) McCann et al. (2015b) Caregiving experience: Experience of caregiving inventory (ECI)
Minds Together	Fitzgeraldson et al. (2024)	Australia	RCT (2)	Caregivers of people with symptoms of depression or anxiety (mean age 50 years)	Unknown	Self‐help and peers	*Quantitative* Quality of life: CarerQol instrument (including CarerQol‐7D and CarerQol‐VAS) Perceived social support (Brief Form of the Perceived Social Support Questionnaire; F‐SozU K‐6) Caregiver perceived burden: ZBI Coping and self‐efficacy: Coping Self‐Efficacy Scale (CSES) Psychological stress: K10
MI.SPOT	Reupert et al. (2020)	Australia	Mixed methods that comprised a pilot uncontrolled single‐group pre‐post design and qualitative interviews (3)	Adult children of people with mental illness or substance use issue (age 18–25 years)	Unknown	Master‐level psychology students	*Quantitative* Wellbeing: Mental Health Continuum Short Form (MHC‐SF) Depression, anxiety and stress: Depression, anxiety and stress scale (DASS‐21) Coping: Coping Orientation to Problems Experienced (COPE) inventory Help seeking behaviour: General Help Seeking Questionnaire Social connectedness: Social Connectedness Scale Mental Health Literacy: Menal Health Literacy Scale Self‐efficacy: General Self‐Efficacy Scale Responsibility: Attributing Responsibility for Parental Care *Qualitative* Acceptability intervention: interview
Supportive care programme (smartphone application)	Minaei‐Moghadam et al. (2024)	Iran	Quasi‐experimental design (3)	Family caregivers (mothers, fathers, child, spouse or siblings) of patients with major depressive disorder (mean age in intervention group was 38 years vs. 40 years in control group)	Unknown	Self‐help and nursing	*Quantitative* Caregiver care burden: Novak and Guest's Caregiver Burden Inventory (CBI) Quality of Life: World Health Organization Quality of Life Questionnaire Short Form (WHOQOL‐BREF)
Web‐based mindfulness intervention (MBI)	Stjernswärd and Hansson (2016, 2018)	Sweden	RCT (2) RCT (2)	Relatives or significant others of a person with mental illness (mean age 54 years) Relatives or significant others of a person with mental illness or somatic illness (mean age 53 years)	Unknown	Self‐help	*Quantitative* Mindfulness: Five Facet Mindfulness Questionnaire (FFMQ) Caregiver burden: CarerQoL‐VAS Self‐compassion: SCS‐SF Perceived stress: PSS Usability: SUS Additional in Stjernswärd and Hansson (2018) Stress and burden: Montgomery‐Borgatta Burden Scale *Qualitative* Usability, confounding factors (other sources of support, negative life events, patient’ health status), and negative effects of training: content analysis additional (SUS) free‐text questions
Web‐based tool	Stjernswärd and Hansson (2014)	Sweden	Explorative mixed methods design (3)	Relatives (parent, child, sibling, partner, ex‐partner or other) of persons with depression (mean age 61 years)	Unknown	Self‐help and peers	*Quantitative* Quality of life: quality of life for depression questionnaire (CarerQol7‐D) Discrimination and stigma: Discrimination and Stigma Scale (DISC‐12) Usability: SUS *Qualitative* Phenomena related to relatives' situations that stand out in the forum, types of social support that are exchanged and social support's potential effects: content analysis text forum

^a^ Level 1: Systematic review or meta‐analysis of RCT's; Level 2: Well‐designed RCT; Level 3: Quasi‐experimental design; Level 4: Single nonexperimental studies; Level 5: Systematic review of descriptive or qualitative studies; Level 6: single descriptive or qualitative studies; Level 7: Opinion of authorities or reports of experts (LoBiondo‐Wood et al. 2019).

Each intervention is briefly described in Table 3, and the theoretical background, description, measurements and intervention elements are presented.

**TABLE 3:** Intervention characteristics.

Intervention	Theoretical background	Description
CCT (Holland Hansen et al. 2021)	Integration of compassion, mindfulness, meditation, psychology, neuroscience and contemplative thinking	CCT is a structured and manualised compassion training programme that focusses on training compassion and kindness for one's own suffering and that of others. The 8‐week course trains participants *in a variety of skills and techniques* for emotional and mental wellbeing. Participants must commit to *class discussions*, meditations and dyadic exercises. They are also asked to meditate daily at home using guided *compassion meditations* that are accessed through a website and to perform informal compassion practices sent by email each week. The CCT instructor (licensed psychologist) strictly follows the manual and receives teaching supervision
DBT Skills group (Wilks et al. 2017)	DBT	Participants follow a 6‐month face‐to‐face DBT *skills group*, which contains mindfulness exercises, home review and *DBT skills* training (*mindfulness, distress tolerance, interpersonal effectiveness and emotion regulation*). Two co‐leaders teach weekly 1.5‐h sessions. The leaders are graduate students or postdoctoral therapists who are part of a DBT team and have undergone DBT training and received supervision
E‐care for caregivers (Bijker et al. 2017)	Psycho‐education and CBT techniques	The intervention has eight modules based on *psycho‐education and CBT* that include the following themes: depression, suicidality, communication, boundary‐setting in caregiving, stress, burnout and self‐care. Each module combines theory with exercises, examples of experiences and videos. Participants choose which module they want to follow. A coach (trained in CBT and receiving supervision) offers personalised feedback and sends supportive reminders. A private internet forum facilitates peer contact
FCSC (Aass et al. 2020)	Calgary family assessment model (CFAM), the Calgary family intervention model (CFIM) and the illness beliefs model (IBM)	FCSC is designed to induce a shift from a deficit‐ or dysfunction‐based family assessment to a strengths‐ and resource‐based conversation that enables families to recognise that each member has a variety of roles. The intervention includes the person suffering from the illness, the family member caring for that person and the mental healthcare professional. Each family has three conversations with the same professional (psychiatric nurse, social worker or social educator with advanced training in, e.g., family assessment and intervention). Conversation subjects include experiences and beliefs in relation to everyday life, family structure, strength and resources, problem‐solving skills, coping strategies and family function. Part of the intervention is family mapping (e.g., making a genogram)
FRPS (Reynolds et al. 2022)	Family systems theory and person‐centred theory	The essence of FRPS is to provide a non‐judgemental, person‐centred space where participants can reflect on the personal impact of having a family member with SMI. Participants attend six 1‐h sessions with their randomly assigned FRPS provider. These providers are volunteers who meet the following criteria: they have experience of supporting family members, have experienced their own recovery process, and are currently at a healthy recovery level. Providers follow a 7‐week training programme and receive supervision during the sessions with the participants. Course content includes peer support, recovery models, recovery process, mental health difficulties, supportive relationships and active peer support
GSH (McCann et al. 2015a, 2015b)	CBT and self‐help principles	The GSH manual has eight modules, and participants complete a module each week. The manual was developed for people with depression, but in this study, it was used for the carers for those patients. All carers receive a weekly supportive call from the researcher (discipline unknown). Module topics include information about depression, value of social contact and physical activity, understanding thought patterns of people with depression, changing thought patterns, solving problems, improving sleep, learning muscle relaxation skills, and coping with stressful setbacks
Minds Together (Fitzgeraldson et al. 2024)	CBT	Minds Together is a web‐based programme consisting of four online modules designed to improve carers' mental health literacy, enhance skills to support the patient–carer relationship, and build their capacity for well‐being and coping. The intervention uses a mixed‐media format, including short videos and podcasts. Participants engage in various activities, such as reading case studies and responding to reflective questions, with access to one module released per week (Fitzgeraldson et al. 2022). Access to a social forum was provided to a subset of participants. In this forum participants can post, comment and react to each other and the researcher ran a weekly ‘happy hour’ to stimulate participants to be online at the same time. Participant got a weekly e‐mail to remind them about this ‘happy hour’ and the researchers posted questions on the forum to encourage discussion
MI.SPOT (Reupert et al. 2020)	Competence enhancement model	MI‐SPOT has various optional components, including a 1‐h weekly synchronous session on different topics (content and aim of MI‐SPOT, illness knowledge, family relationships, stress management, self‐care and help‐seeking strategies) in which a facilitator asks topic‐related questions and stimulates peer support as well as discussion. Every session closes with optional homework, and participants can watch videos from professionals and peers and read informational sheets. Automated questionnaires on weekly topics are provided to enable participants to reflect on their progress. Participants can also schedule a one‐to‐one session, use a peer chat function or upload a video regarding a problematic situation to receive feedback. Finally, participants have weekly mental health check‐ins to monitor their distress level; if the level is high, then they are contacted to receive support. Facilitators are trained master‐psychology students who receive supervision
Supportive care programme via smartphone application (Minaei‐Moghadam et al. 2024)	Care content based on nursing diagnostics	The application's content includes the following topics: nutrition, medication and psychotherapies, sleep hygiene, regulation of activities, definition of depression and related disorders, symptoms of depression, suicidal thoughts and related care, diagnostic measures, communication strategies to improve the patient–carer relationship and electroconvulsive therapy and related care. Options within the application include multimedia care content, medication reminders and a chat section to ask questions of the nurse
Web‐based MBI (Stjernswärd and Hansson 2016, 2018)	Theory of meditation and self‐compassion	This intervention is a web‐based mindfulness programme that can be accessed through a computer, tablet or smartphone. It includes multimedia files, text files, instructions for daily mindfulness that include compassion and self‐compassion exercises, a time log and a private diary. Participants are advised to use the intervention twice a day for 10 min each session 6 days per week for 8 weeks. Weekly email motivation and reminders include technical support's contact information
Web‐based tool (Stjernswärd and Hansson 2014)	Expressive writing and social support	This tool includes a password‐protected web‐based diary to facilitate expressive writing, a user‐only access forum to encourage social support and a psycho‐education module. It promotes communication with the self and others, leading to perspective and empowerment. It stimulates reflection and provides space to release feelings and experiences while obtaining support and advice from others in similar situations

## 4 Results

### 4.1 Selection of Sources of Evidence

See Tables 2 and 3.

### 4.2 Results of Individual Sources of Evidence

#### 4.2.1 Synthesis of Results

The results are structured in three subsections: ‘Description of the Included Articles’ provides an overview of the studies' methodological characteristics, ‘Aim and Focus of the Intervention’ presents the interventions' characteristics and ‘Evaluation and Outcome of the Intervention’ describes the effects and evaluation of the interventions.

### 4.3 Description of the Included Articles

Thirteen articles, which included 11 interventions, met the inclusion criteria. The studies were predominantly quantitative, with six RCTs (Fitzgeraldson et al. 2024; Holland Hansen et al. 2021; McCann et al. 2015a, 2015b; Stjernswärd and Hansson 2016, 2018) and two quasi‐experimental designs (Minaei‐Moghadam et al. 2024; Wilks et al. 2017). Three studies used a mixed‐methods design (Bijker et al. 2017; Reupert et al. 2020; Stjernswärd and Hansson 2014), and two were based on qualitative methods (Aass et al. 2020; Reynolds et al. 2022). The level of evidence (LoBiondo‐Wood et al. 2019) is described in Table 2. Five articles featured a sample of informal caregivers for people with depression (Bijker et al. 2017; McCann et al. 2015a, 2015b; Minaei‐Moghadam et al. 2024; Stjernswärd and Hansson 2014) and the remaining eight had a mixed sample, in which the largest patient group had a depression, anxiety disorder or personality disorder other than BPD (Aass et al. 2020; Fitzgeraldson et al. 2024; Holland Hansen et al. 2021; Reupert et al. 2020; Reynolds et al. 2022; Stjernswärd and Hansson 2016, 2018; Wilks et al. 2017). Almost all interventions focused on the informal caregiver, although one involved a person with the disorder (Aass et al. 2020). Eight articles described professionals as part of the intervention (Aass et al. 2020; Bijker et al. 2017; Holland Hansen et al. 2021; McCann et al. 2015a, 2015b; Minaei‐Moghadam et al. 2024; Reupert et al. 2020; Wilks et al. 2017). Nine articles involved online interventions (Bijker et al. 2017; Fitzgeraldson et al. 2024; McCann et al. 2015a, 2015b; Minaei‐Moghadam et al. 2024; Reupert et al. 2020; Stjernswärd and Hansson 2014, 2016, 2018), three detailed face‐to‐face interventions (Aass et al. 2020; Reynolds et al. 2022; Wilks et al. 2017), and a combination of online and face‐to‐face elements (Holland Hansen et al. 2021). Most studies were conducted in Europe, and professionals in various disciplines performed the interventions. The studies featured describe a variety of quantitative outcome measurements and questionnaires, although most of these measurements were assessed only once. Stress (*n* = 7) and burden (*n* = 7) were the most frequently described outcomes, followed by usability (*n* = 4), quality of life (*n* = 4), resilience (*n* = 3) and self‐compassion (*n* = 3). The outcomes were measured with various questionnaires, and most questionnaires were used once. The SUS was used in four studies that measured the intervention's usability (Bijker et al. 2017; Stjernswärd and Hansson 2014, 2016, 2018). Three of those studies were by the same author (see Table 2).

### 4.4 Aim and Focus of the Interventions

The interventions in the studies focused on different concepts or mechanisms, which were clustered into three categories.

#### 4.4.1 Changing Thoughts and Behaviour

In nine articles, the intervention was intended to change the informal caregiver's thoughts and behaviour about their personal feelings and behaviour in relation to the relative for whom they cared. Six interventions used principles from CBT (Bijker et al. 2017; Fitzgeraldson et al. 2024; McCann et al. 2015a, 2015b; Reupert et al. 2020) or DBT (Wilks et al. 2017); the other interventions were based on different theories such as self‐compassion theory (Holland Hansen et al. 2021; Stjernswärd and Hansson 2016, 2018) or on nursing diagnostics (Minaei‐Moghadam et al. 2024).

Four articles delineated MBIs to enable informal caregivers to accept the illness of their family member's illness and their feelings about that illness (Holland Hansen et al. 2021; Stjernswärd and Hansson 2016, 2018; Wilks et al. 2017). Diaries were included in some interventions (Stjernswärd and Hansson 2014, 2016, 2018) to allow caregivers to reflect on their feelings.

#### 4.4.2 Understanding the Illness

Seven articles mentioned psycho‐education as an element of the intervention (Bijker et al. 2017; McCann et al. 2015a, 2015b; Minaei‐Moghadam et al. 2024; Reupert et al. 2020; Reynolds et al. 2022; Stjernswärd and Hansson 2014). The education was provided online (Bijker et al. 2017; Minaei‐Moghadam et al. 2024; Reupert et al. 2020; Stjernswärd and Hansson 2014), face‐to‐face (Reynolds et al. 2022) or from a self‐help manual (McCann et al. 2015a, 2015b). The psycho‐education comprised different subjects, such as information about the illness, medication, communication and social contact.

#### 4.4.3 Feeling Supported

Six interventions explained peer contact to support informal caregivers and offered online forums or chats (Bijker et al. 2017; Fitzgeraldson et al. 2024; Reupert et al. 2020; Stjernswärd and Hansson 2014) or face‐to‐face contact (Holland Hansen et al. 2021; Reynolds et al. 2022; Wilks et al. 2017). The aim of peer contact was to allow caregivers to ventilate or normalise their feelings, receive support or advice, and acquire feedback on exercises. In six studies, a professional offered the supportive contact (Bijker et al. 2017; McCann et al. 2015a, 2015b; Minaei‐Moghadam et al. 2024; Stjernswärd and Hansson 2016, 2018); two studies described this contact as technical support (Stjernswärd and Hansson 2016, 2018), while the other studies referred to it as psychosocial support. In one study, the supportive contact was provided by both peer and professional (Reupert et al. 2020). One intervention included a medication reminder as a caregiver support tool (Minaei‐Moghadam et al. 2024).

### 4.5 Evaluation and Outcome of the Interventions

The quantitative studies reported different outcomes. *CCT demonstrated* a significant decrease in depression, anxiety, and stress symptoms. Positive effects were observed on overall wellbeing, resilience, self‐compassion and cognitive reappraisal. Perceived stress and emotion suppression (emotion regulation strategy) were reduced. No significant effects were observed regarding awareness or the multidimensional compassion scale score (Holland Hansen et al. 2021). *The DBT skill group* exhibited a significant decrease in emotion dysregulation, interpersonal problems, attitudes towards family members, caregiver strain and perceived stress reactivity, but no significant changes were found on caregiver depression and anxiety (Wilks et al. 2017). *The GSH* intervention experienced a positive effect on caring experiences, a significant reduction in overall negative experiences, and improvement in positive caring experiences (McCann et al.  2015b). The GSH group also showed more growth in resilience than the control group (McCann et al. 2015a). *Minds Together* can help increase perceived social support and quality of life. Adding a social forum to the intervention did not improve outcomes in this study (Fitzgeraldson et al. 2024). *The supportive care programme smartphone application* decreased caregivers' burden and led to a significant increase in quality of life (Minaei‐Moghadam et al. 2024).

Finally, the web‐based MBI participants exhibited a significant decrease in perceived stress. This group witnessed a considerable improvement in mindfulness and self‐compassion as well as in several dimensions (relational, mental health and daily activity problems) of the CarerQoL‐7D. No improvements were seen in the other dimensions (fulfilment, financial, support and physical problems). Most participants rated the programme's usability as good to excellent, but some negative effects were reported regarding training‐related stress. Participants described gradual improvement in the mindfulness group and significant improvements in self‐compassion and perceived stress. There were no significant improvements in caregivers' burden. Although most participants were positive about the training, usability received low scores (Stjernswärd and Hansson 2016, 2018).

Three mixed‐methods studies also offered various quantitative outcomes. *MI.SPOT* significantly reduced participants' depression and stress (Reupert et al. 2020). *E‐care for caregivers* participants initially reported no significant difference in psychological distress compared to the wait‐listed group, although participants noted improvement in their sense of mastery. Wait‐listed subjects reported significantly lower quality of life post‐intervention. The intervention was found to be usable (Bijker et al. 2017). The quantitative outcome in Stjernswärd and Hansson (2014) focussed on quality of life, discrimination and stigma. Participants described relational problems, difficulties coordinating daily activities and caregiving stigma (Stjernswärd and Hansson 2014). Nevertheless, these outcome measurements were used to describe the impact on caregiving rather than the intervention's effect.

The qualitative outcomes in two mixed‐methods studies offered insights into the interventions' user‐friendliness (Bijker et al. 2017) and acceptability (Reupert et al. 2020). MI.SPOT was found to be safe, acceptable and impactful, and participants stated that they felt safe, nurtured, and respected (Reupert et al. 2020). E*‐care for caregivers* (Bijker et al. 2017) was assessed as satisfactory and usable, and the participants reported that the intervention was interesting, informative, educational, effective, useful, practical and helpful in coping and confronting. *The web‐based tool* (Stjernswärd and Hansson 2014) provided qualitative measurements of phenomena related to the relatives' situation and the social support exchanged in the forum. The tool allowed caregivers to explore and reduce their burden by sharing experiences with and receiving support from others in similar circumstances, which may have reduced feelings of social isolation and alienation. A suggestion to improve the forum was to include professional feedback to optimise online support (Stjernswärd and Hansson 2014).

Two studies described only qualitative outcomes. *The FCSC* facilitated conversations regarding topics that family members were previously uncomfortable discussing and supported families as they spoke honestly about emotions or situations concerning everyday family life. Family mapping enabled the development of meaning and possibilities to reveal strengths, competencies and skills. It promoted assessments regarding family composition and how family members function within that family. Family members also mentioned the value of mental health professionals when professionals ask informal caregivers whether they could help and support them (Aass et al. 2020). Participants were also positive about the *FRPS* intervention, reporting that they found it helpful to meet others who could relate to, validate, and normalise their feelings and experiences, which eased social isolation. The significance of the relationship with the provider of the intervention was also acknowledged (Reynolds et al. 2022).

## 5 Discussion

This scoping review provides an extension to previous reviews within the SMI context, by focusing on caregivers for patients with depression, anxiety or personality disorders other than BPD. We located 11 interventions that were designed to support informal caregivers for these specific patient groups. The interventions comprised various elements or mechanisms which we clustered in the following categories: ‘Changing Thoughts and Behaviour’, ‘Understanding the Illness’, and ‘Feeling Supported’. These mechanisms are similar to elements in previous reviews that have focused on psychotic disorders (Chen et al. 2016; Dillinger and Kersun 2020; Holland Hansen et al. 2021; Lobban et al. 2013; Macleod et al. 2011; Yesufu‐Udechuku et al. 2015) or BPD (Guillén et al. 2020; Sutherland et al. 2019). This review extends the findings in previous reviews to provide a broader range of supportive interventions for informal caregivers for people with SMI. The described interventions may be helpful in decreasing informal caregiver burden and strain; however, considering that we identified only 13 relevant articles from our extensive search, we believe that additional research is necessary to explore interventions that support informal caregivers for people with depression, anxiety or personality disorders.

The interventions discussed herein met the needs of informal caregivers, such as the need for information, peer contact (Dillinger and Kersun 2020; Van Husen et al. 2025) and improved interaction skills (Van Husen et al. 2025). Although respite needs have been mentioned in earlier research (Dillinger and Kersun 2020; Macleod et al. 2011; Van Husen et al. 2025), as have the need to be acknowledged as a treatment partner (Dillinger and Kersun 2020), we did not find interventions including these needs.

Several articles mention the relationship between the healthcare professional or peer and the informal caregiver. The intervention strengthened this relationship (Minaei‐Moghadam et al. 2024), and Reynolds et al. (2022) note the healing effect of a safe relationship with the intervention provider. The e‐care intervention (Bijker et al. 2017) reports that some people missed knowing the person who offered feedback, although others favoured anonymous contact.

In patient‐centred care, the relationship between patient and healthcare professional is referred to as a *therapeutic alliance*. The impact of this alliance on the effectiveness of patient‐centred interventions has been stated (Martin et al. 2000). It follows, then, that the alliance or bond with the professional is also of great importance for informal caregivers. Hartley et al. (2020) describe the concept of alliance based on Bording's (1979) definition: an agreement on goals and tasks as well as a therapeutic bond between the therapist and client. This matches the recovery‐focussed research by Burger et al. (2024), which suggests that relational aspects are an important element of collaboration for recovery between persons with SMI, family and professionals; furthermore, Dillinger and Kersun (2020) mention the importance of the level of contact with healthcare professionals. The way in which interventions influence the alliance between informal caregivers and healthcare professionals still remains unclear. Whether and how the alliance affects the intervention's outcome and how the partnership or collaboration influences alliance and outcome has not been systematically researched or published and needs attention in future research.

Although the aim that underscored this scoping review was to map the literature rather than describe the interventions' effectiveness, we paid attention to the experience with and the effects of the interventions. All interventions essentially resulted in positive effects on informal caregivers' psychosocial outcomes (e.g., burden, coping, compassion). We did no methodological quality assessments or comparisons of outcome measurements; hence, the description of the interventions' effects must be seen as an extent of the interventions' description rather than a conclusion regarding which intervention is most effective for supporting informal caregivers.

As noted by Bremmers et al. (2022) and Lobban et al. (2013), the literature regarding the support of informal caregivers offers a variety of outcomes and questionnaires to be used. Consequently, it is difficult to compare intervention effects and describe which intervention should be used in which situation. This heterogeneity was also apparent in this review, where we noticed that interventions were performed by professionals in different disciplines and that there was a wide variety of outcomes and measurements. Further research with either comparable questionnaires or outcomes is necessary to enable a comparison of the interventions' effects.

Additionally, we saw different perspectives on and descriptions of support. On the one hand, support is from an instrumental perspective (e.g., information and tools) while on the other hand, it is relational (e.g., healthcare professionals' initiative to provide support). These contrary perspectives were also mentioned by Stjernswärd and Hansson (2014). To support informal caregivers, it is important to know what support means to the individual informal caregiver and the individual healthcare professionals.

Notably, interventions in this review rarely occurred within daily professional care and were not integrated into the daily care process. This raises questions about how care for informal caregivers is integrated into current psychiatric healthcare and how the interventions are applicable in different healthcare professionals' daily work. Most interventions involved the support of a trained professional (*n* = 8), which indicates that interventions could be implemented by healthcare professionals or be complementary to daily care for people with SMI and their informal caregivers, which is also suggested by (McCann et al. 2015a). Furthermore, support interventions may be used in practice but not published in research. To improve care for informal caregivers, future researchers should clarify how support interventions for informal caregivers can be implemented and garner attention in both psychiatric practice and scientific research.

Finally, previous researchers focused on disorders instead of symptoms, while every person presents with different symptoms, and every informal caregiver has different needs. Future researchers should, therefore, examine the needs in relation to symptoms instead of disorders, which is also underscored by Bremmers et al. (2022). This would provide research opportunities for the nursing profession, as the focus is typically on signs and symptoms rather than illnesses. Furthermore, this demonstrates the importance of relating to the individual caregiver, who has specific personal needs, which Anker‐Hansen et al. (2018) suggest in an article about caregivers for older people with mental health problems who use home care services, as do Dillinger and Kersun (2020) in an article about caregivers for people with first‐episode psychosis. To meet the needs of informal caregivers, an open dialogue about their needs should be the first step in offering support.

### 5.1 Strengths and Limitations

One strength of this review is the focus on the underrepresented diagnoses that meet the SMI definition. The fact that we culled only 13 relevant articles from our sizeable initial search highlights the limited scientific attention regarding support for caregivers for patients who have these illnesses. Another strength of this review is the comprehensive literature search conducted across scientific databases and thorough screening process, which began with a retrieval of 21 720 articles. The article selection process, which had a minimum of two researchers, improves the reliability of the findings. Other than previous reviews, all study designs were included, resulting in a more holistic literature review. An extended search of grey literature could add to this review and provide additional practical tools for healthcare professionals. The comprehensive search can also be seen as a limitation of this study. Because of our extensive search, we identified more articles than were suitable for inclusion in a review article (*n* = 422). Consequently, we needed to determine criteria for reducing the number of articles suitable for inclusion. Given the diversity of professionals in mental health care, we aimed to avoid excluding any professional group from our search. Similarly, we wanted to include all types of research to provide a holistic overview on supportive interventions. Therefore we chose to exclude studies focusing on diagnoses that received substantial attention in recent review articles. Although this second step in the exclusion process was a deviation from protocol and made the methodology more complex, it did not lead to less reliable outcomes. A second limitation of this review is that we included only articles published in English due to the lack of linguistic capacity to read and analyse non‐English publications.

## 6 Conclusion

The aim behind this scoping review was to map the literature on interventions that support informal caregivers for people with SMI. Our specific focus was informal caregivers for people with depression, anxiety disorders or personality disorder other than BPD.

The interventions that were found in the studies seem to decrease informal caregiver burden and strain. However, only 13 relevant articles describing 11 different interventions were identified from our extensive literature search; hence, we emphasise the need for further scientific attention on interventions that support informal caregivers for people with symptoms within the full scope of SMI.

Supporting informal caregivers is complex and requires a patient‐ and family‐centred approach, which professionals should integrate in the care process for persons with SMI and their informal caregivers. Diverse interventions exist that are built on different elements, and outcomes are influenced by various factors. This review offers healthcare professionals multiple options to support informal caregivers for people with SMI. Assessing caregivers' needs should be the first step in supporting this group. Future research is needed to determine how personal and contextual factors influence intervention outcomes and how the different needs of informal caregivers are best addressed.

## 7 Relevance to Clinical Practice

Community‐based mental healthcare provides care to a broad patient population, in which DSM‐5 diagnostics are often comorbid and gradually change. It is therefore important that supportive interventions for informal caregivers are usable and are integrated within the full scope of SMI. As previous researchers have focused on caregivers for people with psychotic disorders, bipolar disorder or BPD, this review extends previous research by focusing on caregivers for people with depression, anxiety or personality disorders other than BPD.

## Funding

The authors have nothing to report.

## Conflicts of Interest

The authors declare no conflicts of interest.

## Supporting information


**Appendix S1:** Search strategy scoping review.

## Data Availability

Data sharing not applicable to this article as no datasets were generated or analysed during the current study.

## References

[inm70236-bib-0001] Aass, L. K. , H. Skundberg‐Kletthagen , A. Schrøder , and Ø. L. Moen . 2020. “Young Adults and Their Families Living With Mental Illness: Evaluation of the Usefulness of Family‐Centered Support Conversations in Community Mental Health Care Settings.” Journal of Family Nursing 26, no. 4: 302–314. 10.1177/1074840720964397. 33095093 PMC7723859

[inm70236-bib-0002] Adelman, R. D. , L. L. Tmanova , D. Delgado , S. Dion , and M. S. Lachs . 2014. “Caregiver Burden: A Clinical Review.” JAMA: The Journal of the American Medical Association 311, no. 10: 1052–1060. 10.1001/jama.2014.304. 24618967

[inm70236-bib-0003] Anker‐Hansen, C. , K. Skovdahl , B. McCormack , and S. Tønnessen . 2018. “The Third Person in the Room: The Needs of Care Partners of Older People in Home Care Services—A Systematic Review From a Person‐Centred Perspective.” Journal of Clinical Nursing 27, no. 7–8: e1309–e1326. 29194850 10.1111/jocn.14205

[inm70236-bib-0004] Anthony, W. A. 1993. “Recovery From Mental Illness: The Guiding Vision of the Mental Health Service System in the 1990s.” Psychosocial Rehabilitation Journal 16, no. 4: 11–23.

[inm70236-bib-0005] Arksey, H. , and L. Malley . 2005. “Scoping Studies: Towards a Methodological Framework.” International Journal of Social Research Methodology 8, no. 1: 19–32. 10.1080/1364557032000119616.

[inm70236-bib-0006] Bijker, L. , A. Kleiboer , H. M. Riper , P. Cuijpers , and T. Donker . 2017. “A Pilot Randomized Controlled Trial of E‐Care for Caregivers: An Internet Intervention for Caregivers of Depressed Patients.” Internet Interventions 9: 88–99. 10.1016/j.invent.2017.06.003. 30135842 PMC6096299

[inm70236-bib-0047] Bordin, E. S. 1979. “The Generalizability of the Psychoanalytic Concept of the Working Alliance.” Psychotherapy: Theory, Research & Practice 16, no. 3: 252–260. 10.1037/h0085885.

[inm70236-bib-0007] Bremmers, L. G. , I. N. Fabbricotti , E. S. Gräler , C. A. Uyl‐de Groot , and L. Hakkaart‐van Roijen . 2022. “Assessing the Impact of Caregiving on Informal Caregivers of Adults With a Mental Disorder in OECD Countries: A Systematic Literature Review of Concepts and Their Respective Questionnaires.” PLoS One 17, no. 7: e0270278. 35802584 10.1371/journal.pone.0270278 PMC9269485

[inm70236-bib-0008] Burger, T. J. , R. M. Van Eck , M. Lachmeijer , et al. 2024. “Perspective Matters in Recovery: The Views of Persons With Severe Mental Illness, Family and Mental Health Professionals on Collaboration During Recovery, a Qualitative Study.” BMC Psychiatry 24: 802. 10.1186/s12888-024-06198-w. 39543545 PMC11566249

[inm70236-bib-0009] Busse, R. , M. Blumel , D. Scheller‐Kreinsen , and A. Zentner . 2010. Tackling Chronic Disease in Europe. European Observatory on Health Systems and Policies.

[inm70236-bib-0010] Chen, L. , J. Liu , J. Zhang , and X. Lu . 2016. “Non‐Pharmacological Interventions for Caregivers of Patients With Schizophrenia: A Meta‐Analysis.” Psychiatry Research 235: 123–127. 26639649 10.1016/j.psychres.2015.11.037

[inm70236-bib-0011] Delespaul, P. H. 2013. “Consensus over de definitie van mensen met een ernstige psychische aandoening (EPA) en hun aantal in Nederland.” Tijdschrift voor Psychiatrie 55, no. 6: 427–438. 23864410

[inm70236-bib-0012] Dillinger, R. L. , and J. M. Kersun . 2020. “Caring for Caregivers: Understanding and Meeting Their Needs in Coping With First Episode Psychosis.” Early Intervention in Psychiatry 14, no. 5: 528–534. 10.1111/eip.12870. 31452318

[inm70236-bib-0013] EUROSTAT . 2020. “Mental Health Care – Psychiatric Hospital Beds.”

[inm70236-bib-0014] Fitzgeraldson, E. , S. Fitzpatrick , J. Dizon , and F. Kay‐Lambkin . 2024. “Evaluating a Targeted Support Program for Mental Health Carers: A Randomised Controlled Trial.” Advances in Mental Health 22: 781–802. 10.1080/18387357.2024.2324103.

[inm70236-bib-0015] Fitzgeraldson, E. , F. Kay‐Lambkin , N. Harding , et al. 2022. “Supports and Interventions for Carers of a Person With Depressive or Anxiety Symptomology: A Systematic Review.” Europe's Journal of Psychology 18: 476–493. 10.5964/ejop.6407. PMC9780730 36605087

[inm70236-bib-0016] Guillén, V. , A. Díaz‐García , A. Mira , et al. 2020. “Interventions for Family Members and Carers of Patients With Borderline Personality Disorder: A Systematic Review.” Family Process 60, no. 1: 134–144. 10.1111/famp.12537. 32304101

[inm70236-bib-0017] Hansen, N. H. , L. Bjerrekær , K. J. Pallesen , L. Juul , and L. O. Fjorback . 2022. “The Effect of Mental Health Interventions on Psychological Distress for Informal Caregivers of People With Mental Illness: A Systematic Review and meta‐Analysis.” Frontiers in Psychiatry 13: 949066. 36276315 10.3389/fpsyt.2022.949066 PMC9583525

[inm70236-bib-0018] Hartley, S. , J. Raphael , K. Lovell , and K. Berry . 2020. “Effective Nurse–Patient Relationships in Mental Health Care: A Systematic Review of Interventions to Improve the Therapeutic Alliance.” International Journal of Nursing Studies 102: 103490. 10.1016/j.ijnurstu.2019.103490. 31862531 PMC7026691

[inm70236-bib-0019] Holland Hansen, N. , L. Juul , K. Pallesen , and L. O. Fjorback . 2021. “Effect of a Compassion Cultivation Training Program for Caregivers of People With Mental Illness in Denmark.” JAMA Network Open 4, no. 3: e211020. 10.1001/jamanetworkopen.2021.1020. 33683334 PMC7941195

[inm70236-bib-0020] Lean, M. , M. Fornells‐Ambrojo , A. Milton , et al. 2019. “Self‐Management Interventions for People With Severe Mental Illness: Systematic Review and meta‐Analysis.” British Journal of Psychiatry 214, no. 5: 260–268. 10.1192/bjp.2019.54. PMC6499726 30898177

[inm70236-bib-0021] Lobban, F. , A. Postlethwaite , D. Glentworth , et al. 2013. “A Systematic Review of Randomised Controlled Trials of Interventions Reporting Outcomes for Relatives of People With Psychosis.” Clinical Psychology Review 33, no. 3: 372–382. 23410719 10.1016/j.cpr.2012.12.004

[inm70236-bib-0022] LoBiondo‐Wood, G. , J. Haber , and M. G. Titler . 2019. “Models and Evidence.” In Evidence‐Based Practice for Nursing and Healthcare Quality Improvement, 24. Elsevier.

[inm70236-bib-0023] Macleod, S. H. , L. Elliott , and R. Brown . 2011. “What Support Can Community Mental Health Nurses Deliver to Carers of People Diagnosed With Schizophrenia? Findings From a Review of the Literature.” International Journal of Nursing Studies 48, no. 1: 100. 10.1016/j.ijnurstu.2010.09.005. 20956000

[inm70236-bib-0024] Martin, D. J. , J. P. Garske , and M. K. Davis . 2000. “Relation of the Therapeutic Alliance With Outcome and Other Variables: A Meta‐Analytic Review.” Journal of Consulting and Clinical Psychology 68: 438–450. 10.1037/0022-006x.68.3.438. 10883561

[inm70236-bib-0025] McCann, T. V. , W. Songprakun , and J. Stephenson . 2015a. “Efficacy of a Self‐Help Manual in Increasing Resilience in Carers of Adults With Depression in Thailand.” International Journal of Mental Health Nursing 25, no. 1: 62–70. 10.1111/inm.12178. 26666688

[inm70236-bib-0026] McCann, T. V. , W. Songprakun , and J. Stephenson . 2015b. “A Randomized Controlled Trial of Guided Self‐Help for Improving the Experience of Caring for Carers of Clients With Depression.” Journal of Advanced Nursing 71, no. 7: 1600–1610. 10.1111/jan.12624. 25656334

[inm70236-bib-0027] Minaei‐Moghadam, S. , Z. S. Manzari , S. Vaghee , and S. Mirhosseini . 2024. “Effectiveness of a Supportive Care Program via a Smartphone Application on the Quality of Life and Care Burden Among Family Caregivers of Patients With Major Depressive Disorder: A Randomized Controlled Trial.” BMC Public Health 24, no. 1: 66. 10.1186/s12889-023-17594-4. 38166907 PMC10762964

[inm70236-bib-0028] National Institute of Mental Health . 2024. Autism Spectrum Disorder. What Is ASD. https://www.nimh.nih.gov/health/topics/autism‐spectrum‐disorders‐asd.

[inm70236-bib-0046] Ouzzani, M., H. Hammady, Z. Fedorowicz, and A. Elmagarmid. 2016. “Rayyan — a Web and Mobile App for Systematic Reviews.” Systematic Reviews 5, 210. 10.1186/s13643-016-0384-4. 27919275 PMC5139140

[inm70236-bib-0029] Peters, M. D. J. , C. M. Godfrey , H. Khalil , P. McInerney , D. Parker , and C. B. Soares . 2015. “Guidance for Conducting Systematic Scoping Reviews.” International Journal of Evidence‐Based Healthcare 13, no. 3: 141–146. 10.1097/XEB.0000000000000050. 26134548

[inm70236-bib-0030] Pharoah, F. , J. J. Mari , J. Rathbone , W. Wong , and C. B. Irving . 2010. “Family Intervention for Schizophrenia.” Cochrane Library, no. 12. 10.1002/14651858.CD000088.pub3.

[inm70236-bib-0031] Reinares, M. , C. M. Bonnín , D. Hidalgo‐Mazzei , J. Sánchez‐Moreno , F. Colom , and E. Vieta . 2016. “The Role of Family Interventions in Bipolar Disorder: A Systematic Review.” Clinical Psychology Review 43: 47–57. 10.1016/j.cpr.2015.11.010. 26691629

[inm70236-bib-0032] Reupert, A. , D. Maybery , C. Bartholomew , et al. 2020. “The Acceptability and Effectiveness of an Online Intervention for Youth With Parents With a Mental Illness and/or Substance Use Issue.” Journal of Adolescent Health 66, no. 5: 551. 10.1016/j.jadohealth.2019.11.309. 32001142

[inm70236-bib-0033] Reynolds, D. , A. Mcmahon , and J. Mcmahon . 2022. “Being Held Through Pain: An Interpretative Phenomenological Analysis of Experiences of Receiving a Peer Support Intervention for Family Members of Individuals With Mental Illness.” Counselling and Psychotherapy Research 22, no. 3: 736. 10.1002/capr.12513.

[inm70236-bib-0034] Spasova, S. , R. Baeten , and B. Vanhercke . 2018. “Challenges in Long‐Term Care in Europe.” Eurohealth 24, no. 4: 7–12.

[inm70236-bib-0035] Stjernswärd, S. , and L. Hansson . 2018. “Effectiveness and Usability of a Web‐Based Mindfulness Intervention for Caregivers of People With Mental or Somatic Illness. A Randomized Controlled Trial.” Internet Interventions 12: 46. 10.1016/j.invent.2018.03.004. 30135768 PMC6096325

[inm70236-bib-0036] Stjernswärd, S. , and L. Hansson . 2016. “Effectiveness and Usability of a Web‐Based Mindfulness Intervention for Families Living With Mental Illness.” Mindfulness 8, no. 3: 751. 10.1007/s12671-016-0653-2. 28515801 PMC5408047

[inm70236-bib-0037] Stjernswärd, S. , and L. Hansson . 2014. “A Web‐Based Supportive Intervention for Families Living With Depression: Content Analysis and Formative Evaluation.” JMIR Research Protocols 3, no. 1: e8. 10.2196/resprot.3051. 24550185 PMC3936281

[inm70236-bib-0038] Sutherland, R. , J. Baker , and S. Prince . 2019. “Support, Interventions and Outcomes for Families/Carers of People With Borderline Personality Disorder: A Systematic Review.” Personality and Mental Health 14: 199–214. 10.1002/pmh.1473. 31887229

[inm70236-bib-0039] Tricco, A. C. , E. Lillie , W. Zarin , et al. 2018. “PRISMA Extension for Scoping Reviews (PRISMA‐ScR): Checklist and Explanation.” Annals of Internal Medicine 169: 467–473. 10.7326/m18-0850. 30178033

[inm70236-bib-0040] Van Husen, G. , T. J. Burger , M. B. De Koning , M. A. S. De Wit , M. W. Segeren , and A. T. F. Beekman . 2025. “Needs of the Network: A Qualitative Study of the Needs of Family Members, Partners and Close Friends of People With a Severe Mental Illness (SMI).” BMC Psychiatry 25: 220. 10.1186/s12888-025-06607-8. 40069728 PMC11895330

[inm70236-bib-0041] Vanner, S. , and R. Keet . 2019. “The Role of Nursing in Community Mental Health.” American Journal of Nursing Studies 1, no. 1: 1002.

[inm70236-bib-0042] WHO . 2022. “Mental Disorders.” https://www.who.int/news‐room/fact‐sheets/detail/mental‐disorders/?gclid=EAIaIQobChMI‐sqEq8H1ggMVJ4poCR1F2APzEAAYASAAEgI‐9_D_BwE.

[inm70236-bib-0043] WHO . 2008. “Policies and Practices for Mental Health in Europe‐Meeting the Challenges.” https://iris.who.int/handle/10665/107366.

[inm70236-bib-0044] Wilks, C. R. , H. Valenstein‐Mah , H. Tran , A. M. M. King , A. Lungu , and M. M. Linehan . 2017. “Dialectical Behavior Therapy Skills for Families of Individuals With Behavioral Disorders: Initial Feasibility and Outcomes.” Cognitive and Behavioral Practice 24: 288–295.

[inm70236-bib-0045] Yesufu‐Udechuku, A. , B. Harrison , E. Mayo‐Wilson , et al. 2015. “Interventions to Improve the Experience of Caring for People With Severe Mental Illness: Systematic Review and meta‐Analysis.” British Journal of Psychiatry 206, no. 4: 268–274. 10.1192/bjp.bp.114.147561. 25833867

