# Supplementary material for: Healthcare Interventions to Support Informal Caregivers of People With Severe Mental Illnesses: A Scoping Review
Source: Int J Ment Health Nurs. 2026 Feb 11;35(1):e70236. doi: 10.1111/inm.70236 (PMC12895140; doi:10.1111/inm.70236)
Supplement: Supplementary file 1 — Appendix S1: Search strategy scoping review. [file INM-35-0-s001.docx]

**Supplement A**

**Search Strategy Scoping Review**

**Pubmed**

(((("family"[MeSH Terms] OR "famil*"[Title] OR "caregivers"[MeSH Terms] OR "caregiver*"[Title] OR "spouse*"[Title] OR "relative*"[Title] OR "dyad*"[Title] OR "husband*"[Title] OR "wife"[Title] OR "wives"[Title] OR "sibling*"[Title] OR "brother*"[Title] OR "sister*"[Title]) AND ("mental illness*"[Title/Abstract] OR "mental disorder*"[Title/Abstract] OR "bipolar disorder*"[Title/Abstract] OR "bipolar disorder"[MeSH Terms] OR "schizophren*"[Title/Abstract] OR "psychot*"[Title/Abstract] OR "psychotic disorders"[MeSH Terms] OR "depres*"[Title/Abstract] OR ("depressive disorder"[MeSH Terms] OR "depression"[MeSH Terms]) OR "depressive disorder"[MeSH Terms] OR "anxiet*"[Title/Abstract] OR "anxiety disorders"[MeSH Terms] OR "Substance-Related Disorders"[MeSH Terms] OR "substance related disorder*"[Title/Abstract] OR "addict*"[Title/Abstract] OR "personality disorder*"[Title/Abstract] OR "personality disorders"[MeSH Terms] OR "forensic*"[Title/Abstract] OR "autistic"[Title/Abstract] OR "autism*"[Title/Abstract] OR "autism spectrum disorder"[MeSH Terms]) AND "support*"[Title/Abstract] AND ("Psychosocial Intervention"[MeSH Terms] OR "intervention*"[Title/Abstract] OR "train*"[Title/Abstract])) NOT ("Child"[MeSH Terms] NOT "Adult"[MeSH Terms])) NOT ("child*"[Title] OR "dementia*"[Title] OR "intensive care"[Title] OR "cancer*"[Title] OR "stroke"[Title])) AND (1993:2024[pdat])

**PsycINFO**

( (DE "Family" OR DE "Biological Family" OR DE "Dysfunctional Family" OR DE "Extended Family" OR DE "Family Background" OR DE "Family History" OR DE "Family Members" OR DE "Family of Origin" OR DE "Family Relations" OR DE "Family Work Relationship" OR DE "Interethnic Family" OR DE "Interracial Family" OR DE "Marriage" OR DE "Military Families" OR DE "Nuclear Family" OR DE "Stepfamily" OR DE "Caregivers" OR TI ( famil* OR caregiver* OR spouse* OR relative* OR dyad* OR husband* OR wife OR wives OR sibling* OR brother* OR sister*)) ) AND ( (DE "Depression (Emotion)" OR DE "Major Depression" OR DE "Anaclitic Depression" OR DE "Dysthymic Disorder" OR DE "Endogenous Depression" OR DE "Late Life Depression" OR DE "Recurrent Depression" OR DE "Treatment Resistant Depression" OR DE "Psychosis" OR DE "Affective Psychosis" OR DE "Alcohol Induced Psychotic Disorders" OR DE "Capgras Syndrome" OR DE "Childhood Onset Psychosis" OR DE "Chronic Psychosis" OR DE "Delusional Disorder" OR DE "Hallucinosis" OR DE "Paranoid Psychosis" OR DE "Schizophrenia" OR DE "Substance Induced Psychotic Disorders" OR DE "Anxiety Disorders" OR DE "Generalized Anxiety Disorder" OR DE "Panic Attack" OR DE "Panic Disorder" OR DE "Phobias" OR DE "Personality Disorders" OR DE "Antisocial Personality Disorder" OR DE "Avoidant Personality Disorder" OR DE "Borderline Personality Disorder" OR DE "Dependent Personality Disorder" OR DE "Histrionic Personality Disorder" OR DE "Narcissistic Personality Disorder" OR DE "Obsessive Compulsive Personality Disorder" OR DE "Paranoid Personality Disorder" OR DE "Passive Aggressive Personality Disorder" OR DE "Sadomasochistic Personality" OR DE "Schizoid Personality Disorder" OR DE "Schizotypal Personality Disorder" OR DE "Autism Spectrum Disorders" OR DE "Autistic Traits" OR TI (“mental illness*” OR “mental disorder*” OR “bipolar disorder*” OR schizophren* OR psychot* OR depres* OR anxiet* OR “substance related disorder*” OR addict* OR “personality disorder*” OR forensic* OR autistic OR autism*) OR AB (“mental illness*” OR “mental disorder*” OR “bipolar disorder*” OR schizophren* OR psychot* OR depres* OR anxiet* OR “substance related disorder*” OR addict* OR “personality disorder*” OR forensic* OR autistic OR autism*)) ) AND ( (TI support* OR AB support*) ) AND ( (DE "Psychosocial Interventions" OR DE "Cognitive Stimulation Therapy" OR TI (intervention* OR train*) OR AB (intervention* OR train*)) NOT TI (child* OR dementia* OR “intensive care” OR cancer* OR stroke) )

**CINAHL**

( ((MH "Family+") OR (MH "Caregivers") OR TI (famil* OR caregiver* OR spouse* OR relative* OR dyad* OR husband* OR wife OR wives OR sibling* OR brother* OR sister*)) ) AND ( ((MH "Bipolar Disorder+") OR (MH "Psychotic Disorders+") OR (MH "Depression+") OR (MH "Anxiety Disorders+") OR (MH "Substance Use Disorders+") OR (MH "Personality Disorders+") OR (MH "Autistic Disorder") OR TI (“mental illness*” OR “mental disorder*” OR “bipolar disorder*” OR schizophren* OR psychot* OR depres* OR anxiet* OR “substance related disorder*” OR addict* OR “personality disorder*” OR forensic* OR autistic OR autism*) OR AB (“mental illness*” OR “mental disorder*” OR “bipolar disorder*” OR schizophren* OR psychot* OR depres* OR anxiet* OR “substance related disorder*” OR addict* OR “personality disorder*” OR forensic* OR autistic OR autism*)) ) AND ( (TI support* OR AB support*) ) AND ( ((MH "Psychosocial Intervention") OR TI (intervention* OR train*) OR AB (intervention* OR train*)) NOT TI (child* OR dementia* OR “intensive care” OR cancer* OR stroke) )

**Cochrane library**

## (famil*:ti OR caregiver*:ti OR spouse*:ti OR relative*:ti OR dyad*:ti OR husband*:ti OR wife:ti OR wives:ti OR sibling*:ti OR brother*:ti OR sister*:ti) AND ((“mental” NEXT illness*):ti,ab,kw OR (“mental” NEXT disorder*):ti,ab,kw OR (“bipolar” NEXT disorder*):ti,ab,kw OR schizophren*:ti,ab,kw OR psychot*:ti,ab,kw OR depres*:ti,ab,kw OR anxiet*:ti,ab,kw OR (“substance related” NEXT disorder*):ti,ab,kw OR addict*:ti,ab,kw OR (“personality” NEXT disorder*):ti,ab,kw OR forensic*:ti,ab,kw OR autistic:ti,ab,kw OR autism*:ti,ab,kw) AND Support*:ti,ab,kw AND (intervention*:ti,ab,kw OR train*:ti,ab,kw) NOT (child*:ti OR dementia*:ti OR (“intensive” NEXT care):ti OR cancer*:ti OR stroke:ti)"

## (famil*:ti OR caregiver*:ti OR spouse*:ti OR relative*:ti OR dyad*:ti OR husband*:ti OR wife:ti OR wives:ti OR sibling*:ti OR brother*:ti OR sister*:ti) AND ((“mental” NEXT illness*):ti,ab,kw OR (“mental” NEXT disorder*):ti,ab,kw OR (“bipolar” NEXT disorder*):ti,ab,kw OR schizophren*:ti,ab,kw OR psychot*:ti,ab,kw OR depres*:ti,ab,kw OR anxiet*:ti,ab,kw OR (“substance related” NEXT disorder*):ti,ab,kw OR addict*:ti,ab,kw OR (“personality” NEXT disorder*):ti,ab,kw OR forensic*:ti,ab,kw OR autistic:ti,ab,kw OR autism*:ti,ab,kw) AND Support*:ti,ab,kw AND (intervention*:ti,ab,kw OR train*:ti,ab,kw) NOT (child*:ti OR dementia*:ti OR (“intensive” NEXT care):ti OR cancer*:ti OR stroke:ti)"

## Embase

## ('family'/exp OR 'caregiver'/exp OR (famil* OR caregiver* OR spouse* OR relative* OR dyad* OR husband* OR wife OR wives OR sibling* OR brother* OR sister*):ti) AND ('psychosis'/exp OR 'depression'/exp OR 'anxiety disorder'/exp OR 'drug dependence'/exp  OR 'personality disorder'/exp OR 'autism'/exp OR  (“mental illness*” OR “mental disorder*” OR “bipolar disorder*” OR schizophren* OR psychot* OR depres* OR anxiet* OR “substance related disorder*” OR addict* OR “personality disorder*” OR forensic* OR autistic OR autism*):ab,ti,kw) AND Support*:ab,ti,kw AND ('psychosocial intervention'/exp OR (intervention* OR train*):ab,ti,kw) NOT ('child'/exp NOT 'adult'/exp) NOT (child*:ti OR dementia*:ti OR “intensive care”:ti OR cancer*:ti OR stroke:ti) NOT 'conference abstract'/it
